# Anthropogenically modified habitats favor bigger and bolder lizards

**DOI:** 10.1002/ece3.7124

**Published:** 2021-01-31

**Authors:** Lachlan Pettit, Gregory P. Brown, Georgia Ward‐Fear, Richard Shine

**Affiliations:** ^1^ School of Life and Environmental Sciences University of Sydney Sydney NSW Australia; ^2^ Department of Biological Sciences Macquarie University Sydney NSW Australia

**Keywords:** intraspecific, niche partitioning, resource subsidy, Varanidae

## Abstract

Anthropogenic activities often create distinctive but discontinuously distributed habitat patches with abundant food but high risk of predation. Such sites can be most effectively utilized by individuals with specific behaviors and morphologies. Thus, a widespread species that contains a diversity of sizes and behavioral types may be pre‐adapted to exploiting such hotspots. In eastern Australia, the giant (to >2 m) lizard *Varanus varius* (lace monitor) utilizes both disturbed (campground) and undisturbed (bushland) habitats. Our surveys of 27 sites show that lizards found in campgrounds tended to be larger and bolder than those in adjacent bushland. This divergence became even more marked after the arrival of a toxic invasive species (the cane toad, *Rhinella marina*) caused high mortality in larger and bolder lizards. Some of the behavioral divergences between campground and bushland lizards may be secondary consequences of differences in body size, but other habitat‐associated divergences in behavior are due to habituation and/or nonrandom mortality.

## Graphical abstract

Lace monitors (*Varanus varius*) are keen exploiters of anthropogenically disturbed habitat; however, only a subset of morphological and behavioral phenotypes in this species favor these artificial environments (i.e., large, bold individuals). The arrival of a toxic invader (*Rhinella marina*) may further exacerbate phenotypic divergences.

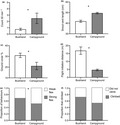

## 1 INTRODUCTION

Humans perturb natural habitats in many ways, often degrading habitat quality to the point where native wildlife is unable to persist (Powers & Jetz, 2019; Sala et al., 2000). However, anthropogenic activities create opportunities as well as costs. One common modification is to create sites with an increased availability of food (resource subsidy) but with a higher risk of predation. For example, agricultural fields attract rats to feast on the crops, but at a greater risk of predation by avian predators (Labuschagne et al., 2016). In many cases, the attributes of the newly created habitat patch differ considerably from that of the ecosystem that they replaced, such that native biota adapted to the previous habitat type will be poorly suited to exploiting the new opportunity. As a result, severe habitat disturbance typically eliminates a high proportion of taxa found in an area (Brooks et al., 2002).

Interestingly, some native taxa (as well as many invasive taxa) thrive in the newly created habitat type (Gangoso et al., 2013). That success is unsurprising for invasive species, many of which exploit anthropogenically modified habitats in their native range and thus do not face the challenge of a niche shift after translocation (Chapple et al., 2011). However, the situation with native taxa is different. The mechanisms that allow that flexibility deserve scrutiny, if we are to understand why anthropogenic activities benefit some native species while disadvantaging others. In particular, which attributes allow a population to exploit a newly created niche, whose use is best‐suited to individuals with phenotypic traits different from those required to exploit the pre‐existing habitat?

One key to success may be phenotypic diversity within the local population. If a population consists of individuals of a wide range of morphologies (e.g., body sizes) and behavioral types (e.g., along the bold‐shy continuum), then some individuals within that population may be well suited to the new opportunity that has arisen. Put simply, ecologically relevant variation among individuals within a population may confer an ecological breadth that pre‐adapts that population to using a novel anthropogenically created habitat type. Why should such variation exist, if it does not adapt individuals to local ecological conditions? One possibility is that the resource hotspot advantages a trait that previously conferred an ecological disadvantage and was maintained only through intense sexual selection. For example, suppose that large body size and bold behavior in males enhance mating opportunities through increased success in male–male combat bouts (e.g., large male American rubyspot damselflies outcompete smaller males for territories and thus increase access to females: Serrano‐Meneses et al., 2007). That situation may result in males evolving to grow to a size at which their maintenance energy requirements can barely be met; that is, the optimal body size from an energetic perspective is smaller than that favored by sexual selection. For example, reproductive success correlates with size in male marine iguanas, but large males suffer the largest mortality when environmental conditions decline (Wikelski & Trillmich, 1997). In such a system, the creation of a novel habitat type that is rich in food resources and most effectively utilized by a large animal might alleviate the energetic disadvantages of large size. If so, a population with a broad range of body sizes, previously maintained by sexual as well as natural selection, might flourish in a newly created resource hotspot.

Our study species—a large species of varanid (monitor) lizard in eastern Australia—is ideal for investigating this situation. These large (to >2 m) lizards are widely distributed through woodland habitats, and campgrounds scattered within that woodland. Attracted by resource subsidies from campers and picnickers, these lizards (*Varanus varius*, “lace monitors” or “goannas” in local parlance) sometimes attain high densities in campgrounds and refuse dumps, and individuals sampled within these highly disturbed open habitats differ in morphology from conspecifics in adjacent bushland (Amir, 2018; Jessop et al., 2012). Sexual size dimorphism in this species is extreme, with adult males averaging around 2.4 times heavier than adult females (5,320 g vs. 2,225 g; Carter, 1992; Guarino, 2002; Kirshner, 2007). In exceptional cases, males attain >14 kg (>6.2 times the average mass of females: Weavers, 1988). That huge body size likely reflects sexually selected advantages to larger males in wrestling matches for dominance and access to matings (Darwin, 1871; Frýdlová & Frynta, 2010). However, a population of lace monitors also contains much smaller individuals; hatchlings (snout–vent length [SVL] 120 mm, 23 g) are ecologically independent and tend to use different habitats and prey items than do adult conspecifics (King & Green, 1999). Thus, the provision of open resource‐rich habitats potentially offers opportunities to goannas of a wide range of sizes. We predicted (based on the ideas above, plus previous studies: Amir, 2018; Jessop et al., 2012) that campground‐dwelling individuals would be larger and also bolder than conspecifics in adjacent bushland regions. Because larger lizards experience high mortality with the invasion of toxic cane toads, *Rhinella marina* (Jolly et al., 2015), we also predicted that the arrival of cane toads (which are most abundant in disturbed habitats: González‐Bernal et al., 2016) would lead to a reduction in mean body sizes and favor monitor lizards that were less bold.

In summary, then, we have a reptilian predator (the lace monitor) and a toxic invader (the cane toad) in a habitat matrix that includes patches of high resource availability (campgrounds) surrounded by large expanses of habitat lacking a resource subsidy (bushland). Surveys of campgrounds and bushland in sites with versus without cane toads allowed us to address three questions: (a) do lizards in campgrounds versus bushland differ in size and boldness?; (b) are habitat‐associated differences in boldness a secondary consequence of differences in mean body size?; and (c) is the distribution of phenotypic traits across that habitat dichotomy affected by the arrival of an invasive species?

## 2 MATERIALS AND METHODS

### 2.1 Study species

**Figure 1:**
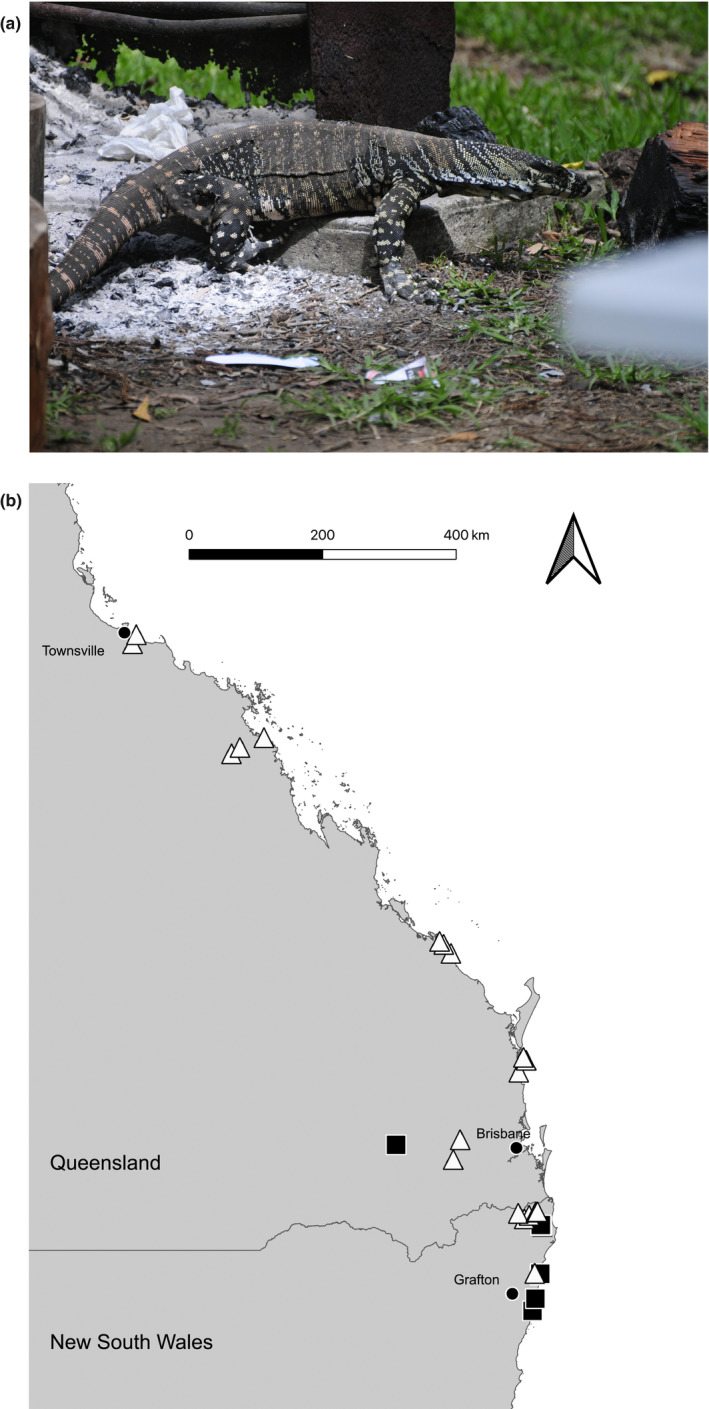
A lace monitor (*Varanus varius*) (a) foraging at a campsite fire pit. Locations of field sites (b) within and beyond the current range of cane toads (*Rhinella marina*) along the east coast of Australia. Filled squares depict toad‐free sites, whereas open triangles show sites that have been colonized by cane toads

Lace monitors are very large (to 14 kg, >2 m) semi‐arboreal lizards with an extensive distribution across eastern Australia (Cogger, 2014; Weavers, 1988). Keystone predators and dietary generalists, lace monitors flexibly exploit seasonal resources, and key in on anthropogenic food subsidies (Jessop et al., 2012; Figure 1a). The longterm numerical impact of toads on lace monitors appears to be minor (especially, compared with impacts on other varanid species: Doody et al., 2009; Pettit et al., In review). Native to Central and South America, cane toads (*Rhinella marina*) were introduced into northeast Australian sugar cane fields in 1935 in an unsuccessful attempt to control insect pests (Zug & Zug, 1979). Once established, toads rapidly spread west through the wet‐dry tropics of Queensland, the Northern Territory and Western Australia (Phillips et al., 2006, 2007; Urban et al., 2007), and slowly extended south along coastal regions of Queensland and New South Wales (Seabrook, 1991). The southern invasion front has expanded through a series of inadvertent and intentional introductions as well as slow‐moving range expansion (van Beurden & Grigg, 1980; Seabrook, 1991). Cane toads have had disastrous impacts on many Australian taxa, especially large‐bodied anurophagous predators that are fatally poisoned if they ingest a toad (Smith & Phillips, 2006).

### 2.2 Study area

We worked at 27 sites along the east coast of Australia (Figure 1b). Each site consisted of a campground and an adjacent 5 km road transect through native bushland. We estimated population sizes of lizards from 10 standardized visual encounter surveys per site (details below). Morphological estimates, habitat characteristics, and behavioral data were gathered from lizards during these surveys and from opportunistic observations. Seven of our sites lacked cane toads, whereas the other 20 had been invaded 1–80 years previously.

### 2.3 Survey methodology

Surveys took place between October 2017 and April 2018 on days with maximum air temperature >23°C. Those five sampling days were split across a minimum of two sampling periods, stratified over time to minimize seasonal biases. Groups of one to three sites were surveyed concurrently for logistical reasons, with surveys of grouped sites, and sites within those groups, randomly ordered to further minimize sampling biases among sites with respect to season and time‐of‐day.

Diurnal surveys for lizards were conducted twice daily between 0800 and 1700 hr AEST. Each visual survey totaled 1‐hr duration, consisting of a 15‐min visual encounter survey on foot in and around the campground, and 45 min of observation by a single observer from a slow‐moving vehicle (20–40 km/hr) along the 5 km road transect through bushland (see Jolly et al., 2016 for detailed transect survey methods). Nocturnal spotlight surveys were performed between 1900 and 2300 hr to confirm the presence or absence of toads via visual confirmation or calling males.

### 2.4 Population sizes of lace monitors

To estimate the relative abundance of lace monitors in campgrounds and bushland at toad‐absent and toad‐present areas, we used mean sightings per 30 min from our 10 visual encounter surveys per habitat per site to account for differential survey effort between habitats (15 min in campgrounds, 45 min in bushland per survey).

### 2.5 Body sizes of lace monitors

A single observer (LP) estimated the SVL of 247 lizards to the nearest 5 cm (Jolly et al., 2016; Lambert et al., 2012). We validated our size estimates for 65 goannas by placing a tape measure next to placid individuals (*N* = 3) or by using ImageJ (Ver. 2.0) to compare standardized photographs of the lateral side of a goanna versus a tape measure placed in the same location and orientation (*N* = 62). Our visual estimates accorded closely with these more precise measurements (ANOVA *R* ^2^ = 0.89 *F*_1,63_ = 491.01 *p* < .0001).

### 2.6 Behavioral measures

To measure the behavioral attributes of wild reptiles, researchers must adapt their scoring systems to the ecology of the species in question (Byrnes et al., 2016; Ward‐Fear et al., 2018). The four behavioral measures listed below are appropriate for assaying lace monitors along a shyness/boldness axis (see Results). We attempted to take data only once from each individual but animals were not marked and thus, some individuals may have been assayed more than once.

### 2.7 Vegetation cover when sighted

An animal's behavioral phenotype can influence how it selects microhabitats, with shyer individuals found in microhabitats that facilitate crypsis (Ward‐Fear et al., 2019). Accordingly, we assessed the percentage of vegetation cover above 30 cm within a 5 m radius surrounding each lizard when first observed (*n* = 244). We then examined associations between vegetation density and lizard behavior in our two habitat contexts (see analysis for details).

### 2.8 Flight‐initiation distance

Flight‐initiation distance (FID) is the distance at which an animal begins to flee from an approaching threat (Cooper & Frederick, 2007), with animals that initiate flight from further away considered more risk‐averse. This offers a robust estimate of risk‐taking behavior in many taxa (Cooper et al., 2015; Samia et al., 2016), even if individuals are only measured once (Putman et al., 2020). We scored flight‐initiation distance (FID) for 231 encounters with lace monitors. As soon as the lizard was sighted, the observer began walking toward the animal at 1 m/s. When the animal responded by fleeing, ascending a tree, or displaying aggression, we placed a mark on the ground and then placed a second mark at the location from which the animal responded. We recorded locations of the two marks using Sightings (v1.1 for iPhone) mobile application software and quantified linear distances between marks to determine the FID. Lizards that allowed approach to within an arm's length were assigned an FID score of 0. We excluded any lizards that fled at a distance greater than 60 m, due to the uncertainty of assigning the flee response specifically to the approach of the observer.

### 2.9 Primary flee response

We scored the behavioral responses of 231 lizards to the approach of an observer as: (a) defend: raising the forebody off the ground, tail‐flicking, and/or hissing, (b) no response (FID = 0), (c) walk, (d) run, or (e) climb. We classified the first three behaviors as “weak flee responses” (i.e., bold behaviors) and the last two behaviors as “strong flee responses” (i.e., shy behaviors).

### 2.10 Proportion that climbed a tree

Lace monitors often climb trees to escape terrestrial threats (Webb, 1994). Following the approach of the observer for the flight‐initiation distance trial, we scored whether or not the lizard fled up a tree (*n* = 230). We considered animals that climbed trees to be more risk‐averse.

#### 2.10.1 Analyses

Although we were unable to repeatedly assay individuals in a range of ecological contexts to test for behavioral syndromes (Sih et al., 2004), our sampling protocol allows us to test for correlated behaviors of individuals, as expected if those behaviors are driven by underlying syndromes (behavioral axes). To explore these relationships, we used nonparametric Spearman analyses to test for correlations between three behavioral variables: (a) association with dense vegetation (% within 5 m radius), (b) flight‐initiation distance, and (c) strength of the flee response.

Next, we tested for associations between lizards that displayed a strong or weak flight response and the habitat in which they were found. We ran a full factorial mixed model with the flight response (categorical: strong flight response or weak flight response), habitat (bushland or campground) and their interaction as predictor variables, and the vegetation density (% cover) surrounding the lizard was seen as a continuous response variable.

We then ran separate models on our six dependent variables (population size, body size, ground cover, flight‐initiation distance, flee response strength, and proportion of lizards that climbed trees) with habitat (categorical; bushland or campground) as the independent variable. Following significant results, we next ran additional mixed models with habitat as a factor, body size (continuous) as a covariate, and their interaction. Finally, we ran full factorial models with factors of habitat (campground vs. bushland) and toad presence (present or absent), and their interaction. Survey day was included as a continuous fixed effect in all models (except for population size analyses, as mean counts were derived from across all survey days), and site was added as a random effect in all models.

Mixed model analyses were run using the GLIMMIX procedure in SAS 9.4 (SAS Institute, Cary NC). Analyses of continuous dependent variables (counts, body size, ground cover when sighted, flight‐initiation distance) used a normal distribution and identity link function. Analyses with a binary dependent variable (proportion that displayed a strong flee response, proportion that climbed trees) were run with a binomial distribution and a logit link function. We visually checked data for normality, and equality of variances was assessed using the Levene test. We applied transformations to data to improve equality of variances where appropriate. We accepted deviations from normality when samples sizes were large (Sokal & Rohlf, 1995).

## 3 RESULTS

### 3.1 Correlations between behavioral variables

**Figure 2:**
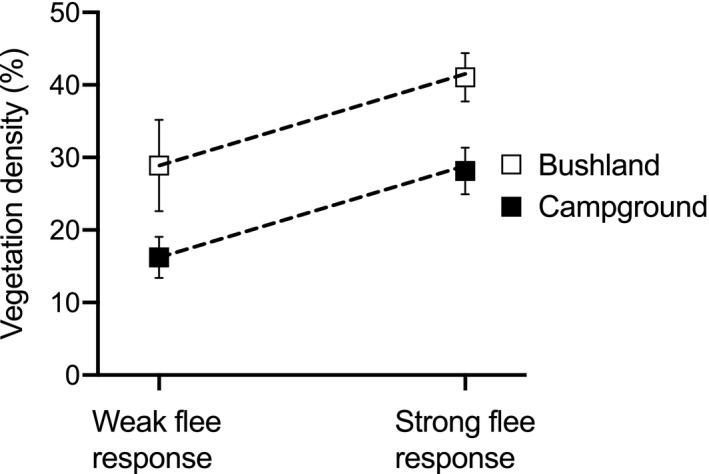
The vegetation density (% cover) in which a lace monitor (*Varanus varius*) was seen as a function of the habitat type (bushland or campground) and the strength of the lizards flight response to an approaching observer

The three behaviors that we scored were positively correlated (all Spearman's tests *p* < .0021). That is, lizards that were spotted in denser vegetation displayed a stronger flee response that was initiated at a greater distance from the observer. These positive correlations support our designations of “bold” or “shy” behaviors across individuals. Additionally, lizards that displayed a strong flight response (running away or climbing a tree in response to an approaching observer) were more likely to be found in dense vegetation (*F*_1,204_ = 4.16, *p* = .043; Figure 2), regardless of being in bushland or campground (habitat x flee response interaction *F*_1,204_ = 0.03, *p* = .86). The main effect of habitat on flight response was also significant, with bushland lizards fleeing from further away (*F*_1,204_ = 5.14, *p* = .025).

### 3.2 Overall comparisons between lizards in campgrounds versus bushland

**Figure 3:**
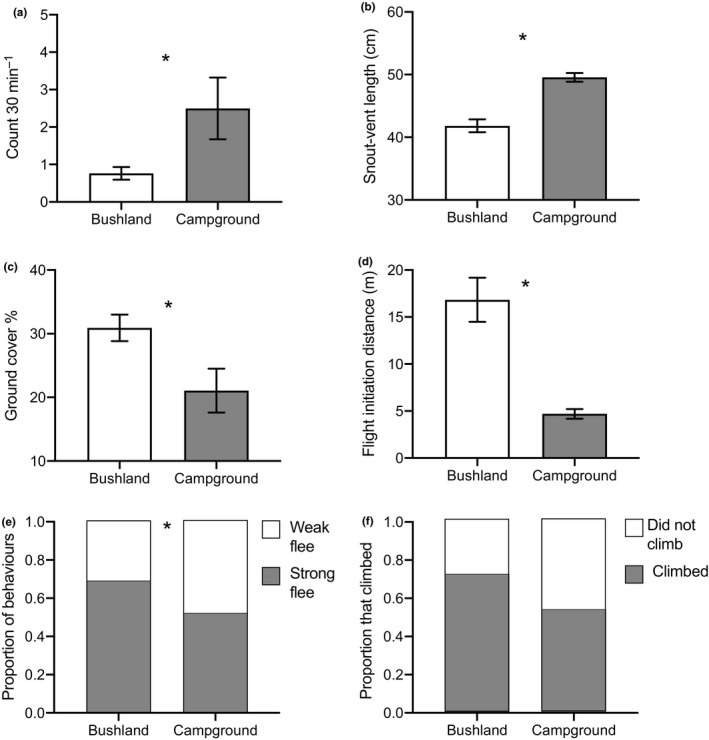
Demographic, morphological, and behavioral characteristics of lace monitors (*Varanus varius*) found in bushland versus campground habitats. An asterisk denotes a significant main effect of habitat. Upper panels depict the mean (±*SE*) (a) count during surveys (number of lizards seen per 30 min) and (b) body sizes (snout–vent lengths) of lizards. Middle panels show the mean (±*SE*) (c) percentage ground cover in which lizards were first seen, and the (d) distance from which a lizard fled from an approaching observer. Bottom panels display the (e) proportion of lizards that exhibited a strong (run or climb) or weak (walk, display aggression, or no response) flee response, and (f) the proportion of lace monitors that fled up a tree. All data are presented graphically in raw form, for ease of interpretation, but some variables had transformations applied prior to statistical analysis

Lace monitors were larger and more abundant in campgrounds than in bushland habitats (Table 1, Figure 3a,b). Despite the more open conditions in campgrounds, lizards in these disturbed areas allowed closer approach before fleeing from an observer and were more likely to ignore the observer or even to exhibit both agonistic behavior toward him rather than fleeing (Table 1, Figure 3c,d,e). Lizards from habitat types were equally likely to climb a tree in response to the approaching observer (Table 1, Figure 3f).

**Table 1:** Results from models exploring the attributes of lace monitors (*Varanus varius*) in bushland (BL) or campgrounds (CG)

Dependent variable	Factors	*F* and *df*	*p* value	Effect direction
Population size	**Habitat**	***F*_1, 26_ = 12.73**	**.0014**	**CG > BL**
Body size	**Habitat**	***F*_1, 222_ = 18.27**	**<.0001**	**CG > BL**
Ground cover	**Habitat**	***F*_1, 219_ = 12.72**	**.0004**	**BL > CG**
Flight‐initiation distance	**Habitat**	***F*_1, 206_ = 48.67**	**<.0001**	**BL > CG**
Flee response strength	**Habitat**	***F*_1, 206_ = 12.31**	**.0006**	**BL > CG**
Proportion that climbed trees	Habitat	*F*_1, 205_ = 3.54	.0612	

Note

Boldface font indicates significant differences between habitat types.

### 3.3 Are behavioral differences between habitats due to differences in mean body size?

There was no significant relationship between the body size of a lizard and the density of vegetation cover in which it was first seen, the distance from which it fled, nor the likelihood of it climbing a tree (Table 2) regardless of the habitat type in which it was found. In both bushland and campgrounds, smaller lizards were more likely to flee (run or climb), whereas larger lizards walked away slowly or allowed the observer to approach (Table 2). Thus, the difference in mean body sizes between campground and bushland lizards was a major driver of the differences in boldness‐associated behaviors shown in Figure 3.

**Table 2:** Results of statistical models exploring how the behavioral attributes of lace monitors (*Varanus varius*) differ with habitat type (bushland [BL] and campgrounds [CG]) and body size

Dependent variable	Factors	*F* and *df*	*p* value	Effect direction
Ground cover	Habitat	*F*_1, 216_ = 3.08	.0808	
	Size	*F*_1, 216_ = 2.17	.1422	
	Habitat × Size	*F*_1, 216_ = 0.78	.3793	
Flight‐initiation distance	Habitat	*F*_1, 204_ = 0.01	.9164	
	Size	*F*_1, 204_ = 2.30	.1313	
	Habitat × Size	*F*_1, 204_ = 3.60	.0592	
Flee response strength	Habitat	*F*_1, 204_ = 0.49	.4847	
	**Size**	***F*_1, 204_ = 4.04**	**.0458**	^a^
	Habitat × Size	*F*_1, 204_ = 0.00	.9538	
Proportion that climbed trees	Habitat	*F*_1, 203_ = 1.97	.1620	
	Size	*F*_1, 203_ = 0.03	.8585	
	Habitat × Size	*F*_1, 203_ = 3.65	.0574	

Note

Boldface font indicates significant main effects and interactions.

^a^ Small lizards exhibited a stronger flee response.

### 3.4 Does the invasion of cane toads affect lizard behavior?

**Figure 4:**
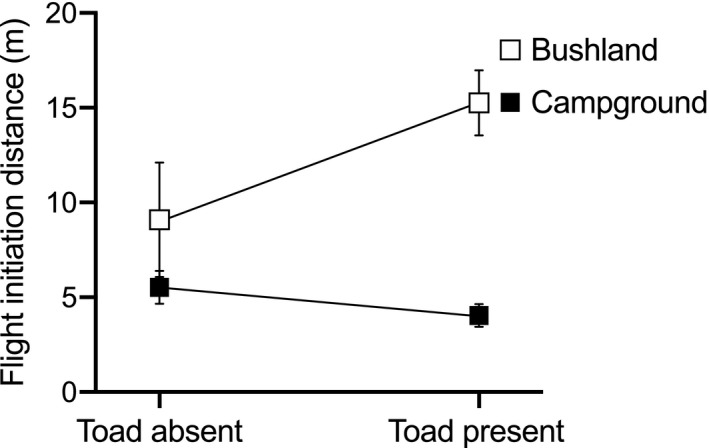
The mean (±*SE*) flight‐initiation distances of lace monitors (*Varanus varius*) found in bushland or campgrounds relative to the presence or absence of cane toads (*Rhinella marina*)

In areas with cane toads, lace monitors were smaller and less abundant, and more wary (stronger flee response) (Table 3). In areas with toads, bushland lizards fled from further away), whereas approach distance was unchanged for campground conspecifics (interaction toad presence*habitat—Table 3, Figure 4). If we split the analysis into habitat types, bushland goannas became shyer (fled from further away) after toads arrived (square root‐transformed; *F*_1,55_ = 4.34, *p* = .041), whereas flight‐initiation distances of campground lizards did not change significantly [log (1 + x) transformed; *F*_1,135_ = 1.32 *p* = .25].

**Table 3:** Results from full factorial models exploring the attributes of lace monitors (*Varanus varius*) in areas differing in habitat (bushland [BL] and campgrounds [CG]) and toad invasion history (toads absent [TA] or toads present [TP])

Dependent variable	Factors	*F* and *df*	*p* value	Effect direction
Population size	**Habitat**	***F*_1,25_ = 16.25**	**.0005**	**CG > BL**
	**Toad status**	***F*_1, 25_ = 4.76**	**.0388**	**TA > TP**
	Habitat × Toad status	*F*_1, 25_ = 2.76	.1091	
Body size	**Habitat**	***F*_1, 221_ = 7.47**	**.0068**	**CG > BL**
	Toad status	*F*_1, 221_ = 3.80	.0525	
	Habitat × Toad status	*F*_1, 221_ = 3.84	.0513	
Ground cover	**Habitat**	***F*_1, 218_ = 9.84**	**.0019**	**BL > CG**
	Toad status	*F*_1, 218_ = 0.00	.9751	
	Habitat × Toad status	*F*_1, 218_ = 0.12	.7246	
Flight‐initiation distance	**Habitat**	***F*_1, 205_ = 15.42**	**<.0001**	**BL > CG**
	Toad status	*F*_1, 205_ = 1.87	.1732	
	**Habitat** × **Toad status**	***F*_1, 205_ = 10.12**	**.0017**	^a^
Flee response strength	**Habitat**	***F*_1, 205_ = 4.79**	**.0298**	**BL > CG**
	Toad status	*F*_1, 205_ = 2.87	.092	
	**Habitat** × **Toad status**	***F*_1, 205_ = 5.08**	**.0253**	^b^
Proportion that climbed trees	Habitat	*F*_1, 204_ = 2.05	.1537	
	Toad status	*F*_1, 204_ = 0.07	.7934	
	Habitat × Toad status	*F*_1, 204_ = 0.01	.9276	

Note

Boldface font indicates significant main effects and interactions.

^a^ Bushland lizards from toad‐present areas fled from the furthest away.

^b^ Bushland lizards from toad‐present areas exhibited the strongest flee response.

## 4 DISCUSSION

Our surveys show that campgrounds support larger monitor lizards than does adjacent native bushland. Previous studies on varanid lizards have shown similar patterns in body size associated with anthropogenically subsidized environments, that may reflect processes such as higher growth rates due to resource subsidies, or intraspecific competition for access to favored sites (Ardiantonio et al., 2018; Jessop et al., 2012; Jolly et al., 2016). Our data are the first to document habitat‐associated differences in abundance, morphology, and behavior of a reptilian predator following a biological invasion. Boldness can be defined as an animal's propensity to engage in risky behavior (Reale et al., 2007; Putman et al., 2020) and hence can be measured as the strength of an individual's response to potential threats (Reale et al., 2007). Bolder individuals may be better able to compete for territory or mates (Reaney & Backwell, 2007), or exploit foraging opportunities in open habitats (Ioannou et al., 2008; Short & Petren, 2008). Hence, boldness may confer strong fitness advantages (Smith & Blumstein, 2008), including higher reproductive success (Ariyomo & Watt, 2012; Reale et al., 2009) and survival (Sinn et al., 2014). However, boldness can also confer costs (e.g., increased predation risk), maintaining variation in behavioral phenotypes within a population (Hulthen et al., 2017).

Although our survey methodology did not allow us to test the same individuals for repeatable behavioral responses across contexts (a requirement to identify behavioral syndromes), significant correlations between our three behavioral measures from each individual are consistent with a spectrum of boldness. Animals that demonstrated the strongest flee response also fled from further away, even though they were concealed in vegetation when first seen (consistent with shy behavior; Ward‐Fear et al., 2019). At the other end of the spectrum, individuals that we found in relatively open habitats often did not move away from us at all. Hence, these behaviors are consistent with boldness [as documented in birds, (Ducatez et al., 2017), mammals (Reale et al., 2000), reptiles (Putman et al., 2020) and fish (Coleman & Wilson, 1998)].

Lizards from bushland habitats fled from further away and adopted tactics (such as climbing trees) consistent with more wary responses. Smaller individuals were less bold than were larger conspecifics, as found also for prey‐handling behavior in this species (Jolly et al., 2016) and for broader dimensions of behavior in another large varanid species from tropical Australia (*V. panoptes*: Ward‐Fear et al., 2018). The behavioral (boldness) difference between campground versus bushland lizards thus was due, at least partly, to the larger average size of campground animals. Interestingly, the invasion of highly toxic cane toads exacerbated some of these habitat‐based divergences between lizards. Below, we explore causal mechanisms underlying these patterns.

Many populations of free‐living animals exhibit substantial variation in individual behavior (consistent patterns of behavior = “personality”: Gosling, 2001), in ways that influence the choice of habitats (Holtmann et al., 2017). For example, we might expect bolder individuals to be better‐suited to disturbed areas like campgrounds, because a highly wary individual in such a site would be able to maintain activity only during the relatively brief periods when no potential threats were evident (Dammhahn & Almeling, 2012). Another mechanism that might generate a correlation between boldness and use of open habitats is habituation: that is, a lizard in such a habitat becomes accustomed to frequent disturbance and so learns to tolerate the approach of a potential threat (such as a human being) without fleeing. Thus, all else being equal, we might expect to see bolder individuals in more open habitats. An individual's body size is likely to affect such habitat partitioning, however, because larger size may render an animal less vulnerable to predators (Urban, 2007), more capable of repelling competitors in competition for a resource (Candolin & Voigt, 2001), and may enable it to ingest a wider range of prey types and sizes (Scharf et al., 2000). Also, size covaries with age, such that a larger animal will likely be older, and thus have had more opportunity to learn the location of local resources (shelters, foraging sites) that facilitate exploitation of a disturbed habitat.

Our data suggest that the behavioral divergence between lizards from campgrounds versus bushland is partly driven by body size effects (larger lizards are bolder, and are more often found in campgrounds) but that campground lizards are bolder than bushland lizards even at the same body size. That correlation might reflect either habituation (campground lizards learn to tolerate frequent disturbance by humans: Samia et al., 2015) or a capacity for individuals to select habitats best‐suited to their own behavioral phenotypes (shy lizards fail to thrive in campgrounds, because they are unable to access resources when bolder conspecifics are present), or to natural selection (e.g., rates of growth and mortality are higher for shy lizards in bushland and for bold lizards in campgrounds). Our data do not allow us to choose between these possibilities. Studies on captive‐raised hatchlings that are the progeny of adults from bushland versus campground habitats would be of great interest.

Frustratingly, we were unable to determine the sex of the lizards that we observed. There are no reliable overt indicators of sex in monitor lizards, even if the animal can be handled, thereby necessitating molecular‐genetics techniques to distinguish between males and females (e.g., Halverson & Spelman, 2002; Ward‐Fear et al., 2018). This was not possible in our study, for logistical reasons. Nonetheless, the marked sexual dimorphism in body sizes within this species (Carter, 1992; Guarino, 2002; Kirshner, 2007) indicates that most of the largest specimens were males. A detailed analysis of a similar‐sized monitor species (*V. panoptes*) showed that in general, males were bolder than females (Ward‐Fear et al., 2018). If the same is true in *V. varius*, then at least part of the habitat‐based divergence in sizes and behavior that we documented may be attributable to sex‐specific habitat partitioning (as reported in several other taxa of reptiles: e.g., Delaney & Warner, 2016; Shine & Wall, 2007). Further work to explore this idea, combining molecular sexing with behavioral observations, would be of great interest. Such a study could also explore the idea (see Introduction) that the large body sizes of adult male lace monitors may confer energetic disadvantages in bushland (where prey are relatively scarce) and hence be maintained by sexual selection rather than natural selection.

The habitat‐based disparities in body sizes and behaviors of lace monitors are of interest not only in their own right, but also because this species is a keystone predator. Recent research has detected multiple trophic shifts in tropical Australia following decimation of monitor (*V. panoptes*) populations by the invasive cane toad (Brown et al., 2013; Doody et al., 2015) and has suggested similar shifts following toad‐induced mortality of *V. varius* (Jolly et al., 2015). The higher abundance, larger size, and bolder behavior of *V. varius* in disturbed (campground) habitats thus may have strong implications for the diverse array of smaller taxa consumed by these giant lizards (Guarino, 2001; Jessop et al., 2010).

A trend for larger, bolder individuals to exploit anthropogenically disturbed habitats may have implications for people as well as for other species, exacerbating conflicts with wildlife. Habituation and boldness of predator species can render them nuisances, leading to dangerous situations for both humans and other animals. For example, bears seeking food are a pervasive threat at campgrounds throughout North America and have caused human fatalities (Rogers, 2011). Coyotes that key in on human subsidies (livestock) demonstrate patterns of bold behavior that also are exhibited by their offspring (via cultural or heritable transmission; Schell et al., 2018). These encounters often lead to culling. Large varanid lizards can inflict serious damage to humans, via aggressive displays (including tail whipping, biting and scratching) and also venom (Fry et al., 2006). The patterns that our study demonstrate are concerning in this respect, and should be considered by managers.

Interestingly, the association between lizard traits and habitat has been reinforced by the recent invasion of cane toads into southeastern Australia. Broadly, we expect mortality due to lethal toxic ingestion of cane toads to fall more heavily on lizards in campgrounds (because toads thrive in disturbed habitats: González‐Bernal et al., 2016) and on larger lizards (because they consume larger toads, and thus ingest more toxin; and because larger goannas evaluate prey less carefully prior to ingesting it: Jolly et al., 2016). So, we might expect toad invasion to disproportionately affect campground lizards, removing the largest and boldest individuals. That would tend to reduce the disparity between campgrounds and bushlands in the attributes of goannas. In practice, however, the disparity was maintained: after toads arrived, bushland lizards fled from further away than did conspecifics in the campground (Table 3, Figure 3b). Indeed, bushland goannas became shyer (fled from further away) after toads, whereas flight‐initiation distances of campground lizards did not change significantly. This pattern may result from behavior‐dependent emigration of lizards from the bushland to the campground after toad‐induced reduction of goanna numbers in campgrounds. That is, the boldest lizards from bushland habitats moved into campgrounds to exploit the newly available opportunities, leaving the shyest individuals as the only ones left in the bushland. That pattern results in an overall decrease in the frequency of bold lizards (as seen in the main effect of toad invasion on strength of the flight response), but no shift within campgrounds in this parameter.

Although the specific type of disturbance and resource subsidy exploited by the lace monitors that we studied are the result of anthropogenic disturbance (i.e., campgrounds), the broad geographic range of lace monitors means that similar “resource hotspots” would have been available even before humans colonized eastern Australia. Thus, for example, monitor lizards have been reported to key in on seasonally or stochastically available food supplies such as fish in drying pools (Shine, 1986; Ward‐Fear et al., 2020) and the eggs and hatchlings of sea turtles (Lei & Booth, 2017). The massive range in both body sizes and behavioral syndromes within a population of large varanid lizards (Ward‐Fear et al., 2018) thus may have allowed these giant reptiles to effectively utilize unpredictable resource‐rich patches within a dynamic habitat mosaic. As humans modified the ecosystems, creating even more extreme spatial and temporal variation in prey availability, species like the lace monitor were ideally placed to take advantage of that new opportunity.

## CONFLICT OF INTEREST

None declared.

## AUTHOR CONTRIBUTIONS

**Lachlan Pettit:** Data curation (lead); formal analysis (lead); funding acquisition (supporting); project administration (lead); software (lead); visualization (lead); writing–original draft (lead). **Greg P. Brown:** Formal analysis (equal). **Georgia Ward‐Fear:** Conceptualization (supporting); funding acquisition (supporting); investigation (supporting); supervision (supporting); writing–review and editing (supporting). **Richard Shine:** Conceptualization (lead); formal analysis (supporting); funding acquisition (lead); investigation (lead); resources (lead); supervision (lead); writing–review and editing (lead).

## ETHICAL APPROVAL

All procedures were approved by the University of Sydney ethics committee (approval 2017/1202) and were carried out in accordance with relevant guidelines and regulations under license from state and federal wildlife agencies.

## Data Availability

Data are housed on the Figshare data repository. https://doi.org/10.6084/m9.figshare.13298732.v1 (Pettit et al., 2020).

## References

[ece37124-bib-0001] Amir, Z. (2018). Relative abundance and risk assessment of lace monitors (*Varanus varius*) on Fraser Island, Queensland: Are monitors habituated to human presence? Biawak, 12, 22–33.

[ece37124-bib-0002] Ardiantonio , Jessop, T. S. , Purwandana, D. , Ciofi, C. , Imansyah, M. J. , Panggur, M. R. , & Ariefiandy, A. (2018). Effects of human activities on Komodo dragons in Komodo National Park. Biodiversity and Conservation, 27(13), 3329–3347. 10.1007/s10531-018-1601-3

[ece37124-bib-0003] Ariyomo, T. O. , & Watt, P. J. (2012). The effect of variation in boldness and aggressiveness on the reproductive success of zebrafish. Animal Behaviour, 83, 41–46.

[ece37124-bib-0005] Brooks, T. M. , Mittermeier, R. A. , Mittermeier, C. G. , Da Fonseca, G. A. B. , Rylands, A. B. , Konstant, W. R. , Flick, P. , Pilgrim, J. , Oldfield, S. , Magin, G. , & Hilton‐Taylor, C. (2002). Habitat loss and extinction in the hotspots of biodiversity. Conservation Biology, 16, 909–923.

[ece37124-bib-0006] Brown, G. P. , Ujvari, B. , Madsen, T. , Shine, R. , & Harwood, J. (2013). Invader impact clarifies the roles of top‐down and bottom‐up effects on tropical snake populations. Functional Ecology, 27, 351–361.

[ece37124-bib-0007] Byrnes, E. E. , Pouca, C. V. , Chambers, S. L. , & Brown, C. (2016). Into the wild: Developing field tests to examine the link between elasmobranch personality and laterality. Behaviour, 153, 1777–1793.

[ece37124-bib-0008] Candolin, U. , & Voigt, H.‐R. (2001). Correlation between male size and territory quality: Consequence of male competition or predation susceptibility? Oikos, 95, 225–230.

[ece37124-bib-0009] Carter, D. B. (1992). Courtship and mating in wild *Varanus varius* (Varanidae: Australia). Memoirs of the Queensland Museum, 29, 33–338.

[ece37124-bib-0010] Chapple, D. G. , Simmonds, S. M. , & Wong, B. (2011). Know when to run, know when to hide: Can behavioral differences explain the divergent invasion success of two sympatric lizards? Ecology and Evolution, 1, 278–289. 22393500 10.1002/ece3.22 PMC3287307

[ece37124-bib-0011] Cogger, H. (2014). Reptiles and amphibians of Australia. CSIRO Publishing.

[ece37124-bib-0012] Coleman, K. , & Wilson, D. S. (1998). Shyness and boldness in pumpkinseed sunfish: Individual differences are context‐specific. Animal Behaviour, 56, 927–936. 9790704 10.1006/anbe.1998.0852

[ece37124-bib-0013] Cooper, W. E. , & Frederick, W. G. (2007). Optimal flight initiation distance. Journal of Theoretical Biology, 244, 59–67. 16949619 10.1016/j.jtbi.2006.07.011

[ece37124-bib-0014] Cooper, W. E. , Samia, D. S. , & Blumstein, D. T. (2015). FEAR, spontaneity, and artifact in economic escape theory: A review and prospectus. Advances in the Study of Behavior, 47, 147–179.

[ece37124-bib-0015] Dammhahn, M. , & Almeling, L. (2012). Is risk taking during foraging a personality trait? A field test for cross‐context consistency in boldness. Animal Behaviour, 84, 1131–1139.

[ece37124-bib-0016] Darwin, C. (1871). The descent of man, and selection in relation to sex. 1871 . J. Murray.

[ece37124-bib-0017] Delaney, D. M. , & Warner, D. A. (2016). Age‐and sex‐specific variations in microhabitat and macrohabitat use in a territorial lizard. Behavioral Ecology and Sociobiology, 70, 981–991.

[ece37124-bib-0018] Doody, J. S. , Green, B. , Rhind, D. , Castellano, C. M. , Sims, R. , & Robinson, T. (2009). Population‐level declines in Australian predators caused by an invasive species. Animal Conservation, 12, 46–53.

[ece37124-bib-0019] Doody, J. S. , Soanes, R. , Castellano, C. M. , Rhind, D. , Green, B. , McHenry, C. R. , & Clulow, S. (2015). Invasive toads shift predator–prey densities in animal communities by removing top predators. Ecology, 96, 2544–2554. 26594710 10.1890/14-1332.1

[ece37124-bib-0020] Ducatez, S. , Audet, J. N. , Rodriguez, J. R. , Kayello, L. , & Lefebvre, L. (2017). Innovativeness and the effects of urbanization on risk‐taking behaviors in wild Barbados birds. Animal Cognition, 20, 33–42. 27287626 10.1007/s10071-016-1007-0

[ece37124-bib-0021] Fry, B. G. , Vidal, N. , Norman, J. A. , Vonk, F. J. , Scheib, H. , Ramjan, S. F. , Kuruppu, S. , Fung, K. , Hedges, S. B. , Richardson, M. K. , Hodgson, W. C. , Ignjatovic, V. , Summerhayes, R. , & Kochva, E. (2006). Early evolution of the venom system in lizards and snakes. Nature, 439, 584–588. 16292255 10.1038/nature04328

[ece37124-bib-0022] Frýdlová, P. , & Frynta, D. (2010). A test of Rensch's rule in varanid lizards. Biological Journal of the Linnean Society, 100, 293–306.

[ece37124-bib-0023] Gangoso, L. , Agudo, R. , Anadón, J. D. , de la Riva, M. , Suleyman, A. S. , Porter, R. , & Donázar, J. A. (2013). Reinventing mutualism between humans and wild fauna: Insights from vultures as ecosystem services providers. Conservation Letters, 6, 172–179.

[ece37124-bib-0024] González‐Bernal, E. , Greenlees, M. J. , Brown, G. P. , & Shine, R. (2016). Toads in the backyard: Why do invasive cane toads (*Rhinella marina*) prefer buildings to bushland? Population Ecology, 58, 293–302.

[ece37124-bib-0025] Gosling, S. D. (2001). From mice to men: What can we learn about personality from animal research? Psychological Bulletin, 127, 45. 11271756 10.1037/0033-2909.127.1.45

[ece37124-bib-0026] Guarino, F. (2001). Diet of a large carnivorous lizard, *Varanus varius* . Wildlife Research, 28, 627–630.

[ece37124-bib-0027] Guarino, F. (2002). Spatial ecology of a large carnivorous lizard, *Varanus varius* (Squamata: Varanidae). Journal of Zoology, 258, 449–457.

[ece37124-bib-0028] Halverson, J. , & Spelman, L. (2002). Sex determination and its role in management In J. B. Murphy , C. Ciofi , C. De La Panouse , & T. Walsh (Eds.), Komodo Dragons: Biology and Conservation. Smithsonian Institution.

[ece37124-bib-0029] Holtmann, B. , Santos, E. S. A. , Lara, C. E. , & Nakagawa, S. (2017). Personality‐matching habitat choice, rather than behavioural plasticity, is a likely driver of a phenotype‐environment covariance. Proceedings of the Royal Society B: Biological Sciences, 284, 20170943. 10.1098/rspb.2017.0943 PMC5647288 28978725

[ece37124-bib-0030] Hulthen, K. , Chapman, B. B. , Nilsson, P. A. , Hansson, L. A. , Skov, C. , Brodersen, J. , Vinterstare, J. , & Bronmark, C. (2017). A predation cost to bold fish in the wild. Scientific Reports, 7, 1239. 28450699 10.1038/s41598-017-01270-w PMC5430796

[ece37124-bib-0031] Ioannou, C. C. , Payne, M. , & Krause, J. (2008). Ecological consequences of the bold‐shy continuum: The effect of predator boldness on prey risk. Oecologia, 157, 177–182. 18481092 10.1007/s00442-008-1058-2

[ece37124-bib-0032] Jessop, T. S. , Smissen, P. , Scheelings, F. , & Dempster, T. (2012). Demographic and phenotypic effects of human mediated trophic subsidy on a large Australian lizard (*Varanus varius*): Meal ticket or last supper? PLoS One, 7, e34069. 22509271 10.1371/journal.pone.0034069 PMC3317928

[ece37124-bib-0033] Jessop, T. , Urlus, J. , Lockwood, T. , & Gillespie, G. (2010). Preying possum: Assessment of the diet of lace monitors (*Varanus varius*) from coastal forests in southeastern Victoria. Biawak, 4, 59–63.

[ece37124-bib-0034] Jolly, C. J. , Shine, R. , & Greenlees, M. J. (2015). The impact of invasive cane toads on native wildlife in southern Australia. Ecology and Evolution, 5, 3879–3894. 26445649 10.1002/ece3.1657 PMC4588653

[ece37124-bib-0035] Jolly, C. J. , Shine, R. , & Greenlees, M. J. (2016). The impacts of a toxic invasive prey species (the cane toad, *Rhinella marina*) on a vulnerable predator (the lace monitor, *Varanus varius*). Biological Invasions, 18, 1499–1509.

[ece37124-bib-0036] King, D. , & Green, B. (1999). Goannas: The biology of varanid lizards. UNSW Press.

[ece37124-bib-0037] Kirshner, D. S. (2007). Multiclutching in captive lace monitors (*Varanus varius*). Mertensiella, 16, 403–421.

[ece37124-bib-0038] Labuschagne, L. , Swanepoel, L. H. , Taylor, P. J. , Belmain, S. R. , & Keith, M. (2016). Are avian predators effective biological control agents for rodent pest management in agricultural systems? Biological Control, 101, 94–102.

[ece37124-bib-0039] Lambert, M. R. , Yasuda, C. M. , & Todd, B. D. (2012). Evaluation of a photographic technique for estimating body size in lizards from a distance. Herpetological Conservation and Biology, 7, 83–88.

[ece37124-bib-0040] Lei, J. , & Booth, D. T. (2017). Who are the important predators of sea turtle nests at Wreck Rock beach? PeerJ, 5, e3515. 28674666 10.7717/peerj.3515 PMC5494172

[ece37124-bib-0041] Pettit, L. , Brown, G. , Ward‐Fear, G. , & Shine, R. (2020). Data from: Anthropogenically modified habitats favour bigger and bolder lizards (Version 1) Figshare. 10.6084/m9.figshare.13298732.v1 PMC7882987 33613991

[ece37124-bib-0042] Pettit, L. , Ward‐Fear, G. , & Shine, R. (In review) Divergent long‐term impacts of lethally toxic cane toads (Rhinella marina) on two species of apex predators (monitor lizards, Varanus spp.). 10.1371/journal.pone.0254032 PMC8297793 34292946

[ece37124-bib-0043] Phillips, B. L. , Brown, G. P. , Greenlees, M. , Webb, J. K. , & Shine, R. (2007). Rapid expansion of the cane toad (*Bufo marinus*) invasion front in tropical Australia. Austral Ecology, 32, 169–176.

[ece37124-bib-0044] Phillips, B. L. , Brown, G. P. , Webb, J. K. , & Shine, R. (2006). Invasion and the evolution of speed in toads. Nature, 439, 803. 16482148 10.1038/439803a

[ece37124-bib-0045] Powers, R. P. , & Jetz, W. (2019). Global habitat loss and extinction risk of terrestrial vertebrates under future land‐use‐change scenarios. Nature Climate Change, 9, 323–329.

[ece37124-bib-0100] Putman, B. J. , Pauly, G. B. , & Blumstein, D. T. (2000). Urban invaders are not bold risk‐takers: A study of three invasive lizards in Southern California. Current Zoology. 10.1093/cz/zoaa015 PMC7769584 33391365

[ece37124-bib-0046] Reale, D. , Gallant, B. Y. , Leblanc, M. , & Festa‐Bianchet, M. (2000). Consistency of temperament in bighorn ewes and correlates with behaviour and life history. Animal Behavior, 60, 589–597. 10.1006/anbe.2000.1530 11082229

[ece37124-bib-0047] Reale, D. , Martin, J. , Coltman, D. W. , Poissant, J. , & Festa‐Bianchet, M. (2009). Male personality, life‐history strategies and reproductive success in a promiscuous mammal. Journal of Evolutionary Biology, 22, 1599–1607. 19555442 10.1111/j.1420-9101.2009.01781.x

[ece37124-bib-0048] Reale, D. , Reader, S. M. , Sol, D. , McDougall, P. T. , & Dingemanse, N. J. (2007). Integrating animal temperament within ecology and evolution. Biological Review Cambridge Philosophical Society, 82, 291–318. 10.1111/j.1469-185X.2007.00010.x 17437562

[ece37124-bib-0049] Reaney, L. T. , & Backwell, P. R. Y. (2007). Risk‐taking behavior predicts aggression and mating success in a fiddler crab. Behavioral Ecology, 18, 521–525.

[ece37124-bib-0050] Rogers, L. (2011). Does diversionary feeding create nuisance bears and jeopardize public safety? Human‐Wildlife Interactions, 5, 287–295.

[ece37124-bib-0051] Sala, O. E. , Chapin, F. S., 3rd , Armesto, J. J. , Berlow, E. , Bloomfield, J. , Dirzo, R. , Huber‐Sanwald, E. , Huenneke, L. F. , Jackson, R. B. , Kinzig, A. , Leemans, R. , Lodge, D. M. , Mooney, H. A. , Oesterheld, M. , Poff, N. L. , Sykes, M. T. , Walker, B. H. , Walker, M. , & Wall, D. H. (2000). Global biodiversity scenarios for the year 2100. Science, 287, 1770–1774. 10710299 10.1126/science.287.5459.1770

[ece37124-bib-0052] Samia, D. S. , Blumstein, D. T. , Stankowich, T. , & Cooper, W. E. Jr (2016). Fifty years of chasing lizards: New insights advance optimal escape theory. Biological Review Cambridge Philosophical Society, 91, 349–366. 10.1111/brv.12173 25620002

[ece37124-bib-0053] Samia, D. S. , Nakagawa, S. , Nomura, F. , Rangel, T. F. , & Blumstein, D. T. (2015). Increased tolerance to humans among disturbed wildlife. Nature Communications, 6, 8877 10.1038/ncomms9877 PMC4660219 26568451

[ece37124-bib-0054] Scharf, F. S. , Juanes, F. , & Rountree, R. A. (2000). Predator size‐prey size relationships of marine fish predators: Interspecific variation and effects of ontogeny and body size on trophic‐niche breadth. Marine Ecology Progress Series, 208, 229–248. 10.3354/meps208229

[ece37124-bib-0055] Schell, C. J. , Young, J. K. , Lonsdorf, E. V. , Santymire, R. M. , & Mateo, J. M. (2018). Parental habituation to human disturbance over time reduces fear of humans in coyote offspring. Ecology and Evolution, 8, 12965–12980. 10.1002/ece3.4741 30619597 PMC6308887

[ece37124-bib-0056] Seabrook, W. (1991). Range expansion of the introduced cane toad *Bufo marinus* in New South Wales. Australian Zoologist, 27, 58–62.

[ece37124-bib-0057] Serrano‐Meneses, M. A. , Córdoba‐Aguilar, A. , Méndez, V. , Layen, S. J. , & Székely, T. (2007). Sexual size dimorphism in the American rubyspot: Male body size predicts male competition and mating success. Animal Behaviour, 73, 987–997. 10.1016/j.anbehav.2006.08.012

[ece37124-bib-0058] Shine, R. (1986). Food habits, habitats and reproductive biology of four sympatric species of varanid lizards in tropical Australia. Herpetologica, 1986, 346–360.

[ece37124-bib-0059] Shine, R. , & Wall, M. (2007). Why is intraspecific niche partitioning more common in snakes than in lizards? In S. M. Reilly , L. D. McBrayer , & D. B. Miles (Eds.), Lizard ecology. The evolutionary consequences of foraging mode (pp. 173–208). Cambridge University Press.

[ece37124-bib-0060] Short, K. H. , & Petren, K. (2008). Boldness underlies foraging success of invasive *Lepidodactylus lugubris* geckos in the human landscape. Animal Behaviour, 76, 429–437. 10.1016/j.anbehav.2008.04.008

[ece37124-bib-0061] Sih, A. , Bell, A. M. , Chadwick Johnson, J. , & Ziemba, R. E. (2004). Behavioral syndromes: An ecological and evolutionary overview. Trends in Ecology and Evolution, 19, 372–378. 10.1016/j.tree.2004.04.009 16701288

[ece37124-bib-0062] Sinn, D. L. , Cawthen, L. , Jones, S. M. , Pukk, C. , & Jones, M. E. (2014). Boldness towards novelty and translocation success in captive‐raised, orphaned Tasmanian devils. Zoo Biology, 33, 36–48. 10.1002/zoo.21108 24375492

[ece37124-bib-0063] Smith, B. R. , & Blumstein, D. T. (2008). Fitness consequences of personality: A meta‐analysis. Behavioral Ecology, 19, 448–455. 10.1093/beheco/arm144

[ece37124-bib-0064] Smith, J. , & Phillips, B. (2006). Toxic tucker: The potential impact of Cane Toads on Australian reptiles. Pacific Conservation Biology, 12, 40–49. 10.1071/PC060040

[ece37124-bib-0065] Sokal, R. R. , & Rohlf, F. J. (1995). Biometry: The principles and practice of statistics in biological research. W.H. Freeman.

[ece37124-bib-0066] Urban, M. C. (2007). The growth–predation risk trade‐off under a growing gape‐limited predation threat. Ecology, 88, 2587–2597. 10.1890/06-1946.1 18027761

[ece37124-bib-0067] Urban, M. C. , Phillips, B. L. , Skelly, D. K. , & Shine, R. (2007). The cane toad's (*Chaunus [Bufo] marinus*) increasing ability to invade Australia is revealed by a dynamically updated range model. Proceedings of the Royal Society B: Biological Sciences, 274, 1413–1419. 10.1098/rspb.2007.0114 PMC2176198 17389221

[ece37124-bib-0068] van Beurden, E. K. , & Grigg, G. C. (1980). An isolated and expanding population of the introduced toad *Bufo marinus* in New South Wales. Wildlife Research, 7, 305–310. 10.1071/WR9800305

[ece37124-bib-0069] Ward‐Fear, G. , Brown, G. P. , Pearson, D. J. , West, A. , Rollins, L. A. , & Shine, R. (2018). The ecological and life history correlates of boldness in free‐ranging lizards. Ecosphere, 9, e02125 10.1002/ecs2.2125

[ece37124-bib-0070] Ward‐Fear, G. , Brown, G. P. , & Shine, R. (2020). Within‐population variation in ecological traits: Implications for vulnerability and impact of imperilled keystone predators. Ecosphere, 11, e03136.

[ece37124-bib-0071] Ward‐Fear, G. , Rangers, B. , Pearson, D. , Bruton, M. , & Shine, R. (2019). Sharper eyes see shyer lizards: Collaboration with indigenous peoples can alter the outcomes of conservation research. Conservation Letters, 12, e12643 10.1111/conl.12643

[ece37124-bib-0072] Weavers, B. W. (1988). Vital statistics of the lace monitor lizard (*Varanus varius*) in south‐eastern Australia. Victorian Naturalist, 105, 142–145.

[ece37124-bib-0073] Webb, J. (1994). Observation of three dingoes killing a large lace monitor (*Varanus varius*). Australian Mammalogy, 19, 55–56.

[ece37124-bib-0074] Wikelski, M. , & Trillmich, F. (1997). Body size and sexual size dimorphism in marine iguanas fluctuate as a result of opposing natural and sexual selection: An island comparison. Evolution, 51, 922–936. 10.1111/j.1558-5646.1997.tb03673.x 28568579

[ece37124-bib-0075] Zug, G. R. , & Zug, P. B. (1979). The marine toad, Bufo marinus: A natural history resume of native populations. Smithsonian Institution Press.

